# Methylome-wide association studies and epigenetic biomarker development for 133 mass spectrometry-assessed circulating proteins in 14,671 Generation Scotland participants

**DOI:** 10.1186/s13059-025-03892-0

**Published:** 2025-12-08

**Authors:** Josephine A. Robertson, Jakub Bajzik, Spyros Vernardis, Aleksandra D. Chybowska, Daniel L. McCartney, Arturas Grauslys, Jure Mur, Hannah M. Smith, Archie Campbell, Camilla Drake, Hannah Grant, Jamie Pearce, Tom C. Russ, Poppy Adkin, Matthew White, Charles Brigden, Christoph B. Messner, David J. Porteous, Caroline Hayward, Simon R. Cox, Aleksej Zelezniak, Markus Ralser, Matthew R. Robinson, Riccardo E. Marioni

**Affiliations:** 1 Institute of Genetics and Cancer, University of Edinburgh, Edinburgh, UK; 2 Institute of Science and Technology, Vienna, Austria; 3 Molecular Biology of Metabolism Laboratory, The Francis Crick Institute, London, UK; 4 Eliptica Limited, The London Cancer Hub, Cotswold Road, Sutton, London, UK; 5 MRC Human Genetics Unit, Institute of Genetics and Cancer, University of Edinburgh, Edinburgh, UK; 6 Centre for Research on Environment, Society and Health, School of Geosciences, University of Edinburgh, Edinburgh, UK; 7 Division of Psychiatry, Centre for Clinical Brain Sciences, University of Edinburgh, Edinburgh, UK; 8 Alzheimer Scotland Dementia Research Centre, Department of Psychology, University of Edinburgh, Edinburgh, UK; 9 Medical Research Council Clinical Trials Unit, University College London, London, UK; 10 Department of Psychology, The Lothian Birth Cohorts, University of Edinburgh, Edinburgh, UK; 11 Department of Biochemistry, Charité Universitätsmedizin Berlin, Berlin, Germany; 12 Centre for Medical Informatics, Usher Institute, University of Edinburgh, Edinburgh, UK; 13 Precision Proteomics Center, Swiss Institute of Allergy and Asthma Research, University of Zurich, Zurich, Switzerland; 14 Randall Centre for Cell & Molecular Biophysics, King’s College London, New Hunt’s House, Guy’s Campus, London, SE1 1UL UK; 15 Department of Biology and Biological Engineering, Chalmers University of Technology, Kemivägen 10, Gothenburg, SE-412 96 Sweden; 16 Institute of Biotechnology, Life Sciences Centre, Vilnius University, Sauletekio al. 7, Vilnius, LT10257 Lithuania

**Keywords:** Epigenetics, Proteomics, Cardiovascular disease, Biomarkers

## Abstract

**Background:**

DNA methylation (DNAm) can regulate gene expression, and its genome-wide patterns (epigenetic scores or EpiScores) can act as biomarkers for complex traits. The relative stability of methylation profiles may enable better assessment of chronic exposures compared to single time-point protein measures. We present the first large-scale epigenetic study of the highly-abundant serum proteome measured via ultra-high throughput mass spectrometry in 14,671 samples from the Generation Scotland cohort. We further demonstrate the first large-scale comparison of protein EpiScores and their respective proteins as predictors of incident cardiovascular disease.

**Results:**

Marginal epigenome-wide association models, adjusting for age, sex, measurement batch, estimated white cell proportions, BMI, smoking and methylation principal components, reveal 15,855 significant CpG – protein associations across 125 of 133 proteins P_Bonferroni_ < 2.71 × 10^-10^. Bayesian epigenome-wide association studies of the same 133 proteins reveal 697 CpG-Protein associations (posterior inclusion probability > 0.95). 112 protein EpiScores correlate significantly with their respective protein in a holdout test-set. Of these, sixteen associate significantly with incident all-cause cardiovascular disease (N_events_=191) compared to one measured protein.

**Conclusions:**

We highlight a complex interplay between the blood-based methylome and proteome. Importantly, we show that protein EpiScores correlate with measured proteins and demonstrate that the, as-yet understudied, high-abundance proteome may yield clinically relevant biomarkers. The protein EpiScores demonstrate more significant associations with cardiovascular disease than directly measured proteins, suggesting their potential as clinical biomarkers for monitoring or predicting disease risk. We suggest that biomarker development could be enhanced by the consideration of protein EpiScores alongside measured proteins.

**Supplementary Information:**

The online version contains supplementary material available at 10.1186/s13059-025-03892-0.

## Background

Blood-based protein measurements are under increasing focus for the development of biomarkers of morbidity and mortality [1, 2], with evidence that protein prediction models outperform models using clinical information [2]. In addition to genetic influences, a complex interplay exists between the proteome and other omics layers, such as the methylome [3]. The methylome describes the pattern of genome-wide DNA methylation (DNAm), the addition of a methyl group to cytosine nucleotides, most commonly occurring at cytosines which precede a guanine (CpG sites) acting to regulate transcription [4]. Epigenome-wide association studies can help elucidate the relationship between DNAm and the circulating proteome, providing greater insight into the latter’s regulation and the potential role of various environmental, biological and lifestyle factors on health.

The use of DNAm-based proxies for complex traits, including protein levels and health outcomes, represents an expanding field of research [5]. Where proteins are concerned, these proxies, which we call epigenetic scores (EpiScores), have been shown to display a more stable longitudinal measurement [6], likely reflecting cumulative and sustained impacts of environmental effects and biological changes [7]. This property is key to the promise of EpiScores as biomarkers and tools for risk prediction, stratification and precision medicine. Research thus far has demonstrated that EpiScores can track proteomic markers to enhance our understanding of the impact of chronic inflammation on both cardiovascular and neurological health [8, 9]; highlight novel associations with incident disease [10]; and augment prediction models for disease, offering measurable improvement in prediction over and above traditional risk factors [11]. Therefore, in the search for disease biomarkers to enhance risk prediction and monitor interventions to improve outcomes, both epigenetic and proteomic biomarkers should be considered.

Studies of the circulating proteome have predominantly captured lower abundant signalling and tissue-leakage proteins, using multiplexed antibody or aptamer-based assays, such as the Olink^®^ and SomaScan platforms, which capture up to ~11,000 proteins. These targeted approaches navigate the challenges of the wide dynamic range of the human proteome and can quantify low-abundant proteins, many of which are potential biomarkers. However, quantification of high-abundance proteins, which constitute 99% of total protein mass in blood [12], using these assays is challenging due to the presence of multiple isoforms and saturation of affinity reagents, limiting the dynamic range of measurement [13]. In contrast to tissue leakage or signalling proteins, these proteins mostly function in processes occurring within blood, such as nutrient transport, innate immunity, or coagulation. Such proteins are well quantified using mass spectrometry (MS) [14] and data acquisition can be untargeted, independent of current paradigms or knowledge [12].

**Fig. 1:**
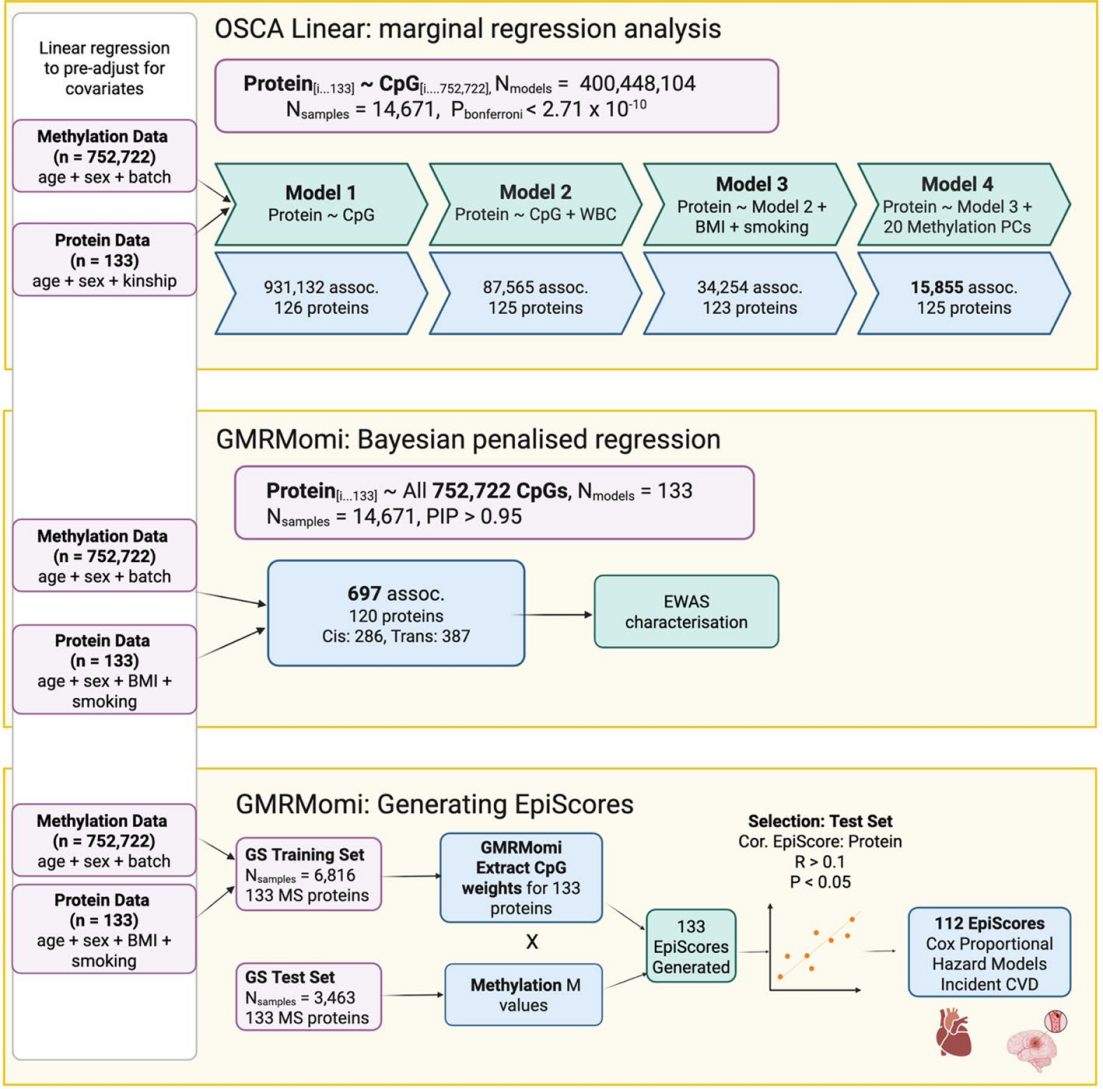
Overview of EWAS and EpiScore workflow and results. For OSCA linear marginal regression analysis, each CpG is modelled individually for every protein within each model. For GMRMomi Bayesian penalised regression, all CpGs are modelled jointly. The Bayesian approach was subsequently used to identify lead CpGs and for the generation of protein EpiScores. WBC = estimated white blood cell proportions; BMI = log transformation of body mass index (kg/m^2^); smoking = log transformation of smoking pack-years (+ 1); PCs = Principal Components; PIP = posterior inclusion probability. Created in BioRender, Marioni, R. (2025) https://BioRender.com/q80a293

In this study, we conduct the first large-scale epigenome-wide association studies of the serum proteome as measured by mass spectrometry, to uncover novel associations between DNAm and proteins. Further, in independent data subsets, we train protein EpiScores and then test their associations with incident cardiovascular disease. We then provide the first large-scale analysis of both measured proteins and protein EpiScores in association with incident cardiovascular disease. Figure 1 illustrates the project overview.

## Results

### EWAS

Marginal linear regression models were run using OmicS-data-based Complex trait Analysis (OSCA, v0.46.1) [15]. In these models each CpG was independently regressed on protein level, yielding 15,855 significant CpG – protein associations across 125 of the 133 uniquely mapped proteins (P_Bonferroni_ < 2.71 × 10^− 10^), with a median genomic inflation factor of 1.17 (Additional file 1: Tables S1 & S2). These models do not account for inter-probe correlations or attempt to fine-map the findings. Consequently, we applied a joint and conditional Bayesian regression approach (Bayesian Grouped Mixture of Regressions Model for analysing OMIcs data, GMRMomi) [16, 17]. This resulted in 697 CpG – protein associations (PIP >0.95) for 120 of 133 uniquely mapped proteins, involving 457 unique CpGs. 286 of these were *cis* associations (CpG within 1 Mb of the transcription start-site (TSS) of the associated protein’s gene) and 387 were *trans* associations (CpG more than 1 Mb outside of the associated protein’s TSS or on a different chromosome) (Additional file 1: TableS3). 298 of the 457 CpGs map to 233 unique genes.

The Bayesian approach identified 505 of the protein-CpG associations (for 115 unique proteins and 333 unique CpGs) found with the OSCA approach (See Additional file 1: Table S4). The direction of the effect was consistent across all loci and there was a Pearson correlation of 0.94 in their effect sizes. Of the remaining 192 associations found via GMRMomi, 67 were present at *P* < 3.6 × 10^− 8^ (epigenome-wide significance threshold) [18], and an additional 119 present at *P* < 0.05 in the OSCA analyses.

### Epigenetic architecture of the lead findings from the Bayesian EWAS

**Fig. 2:**
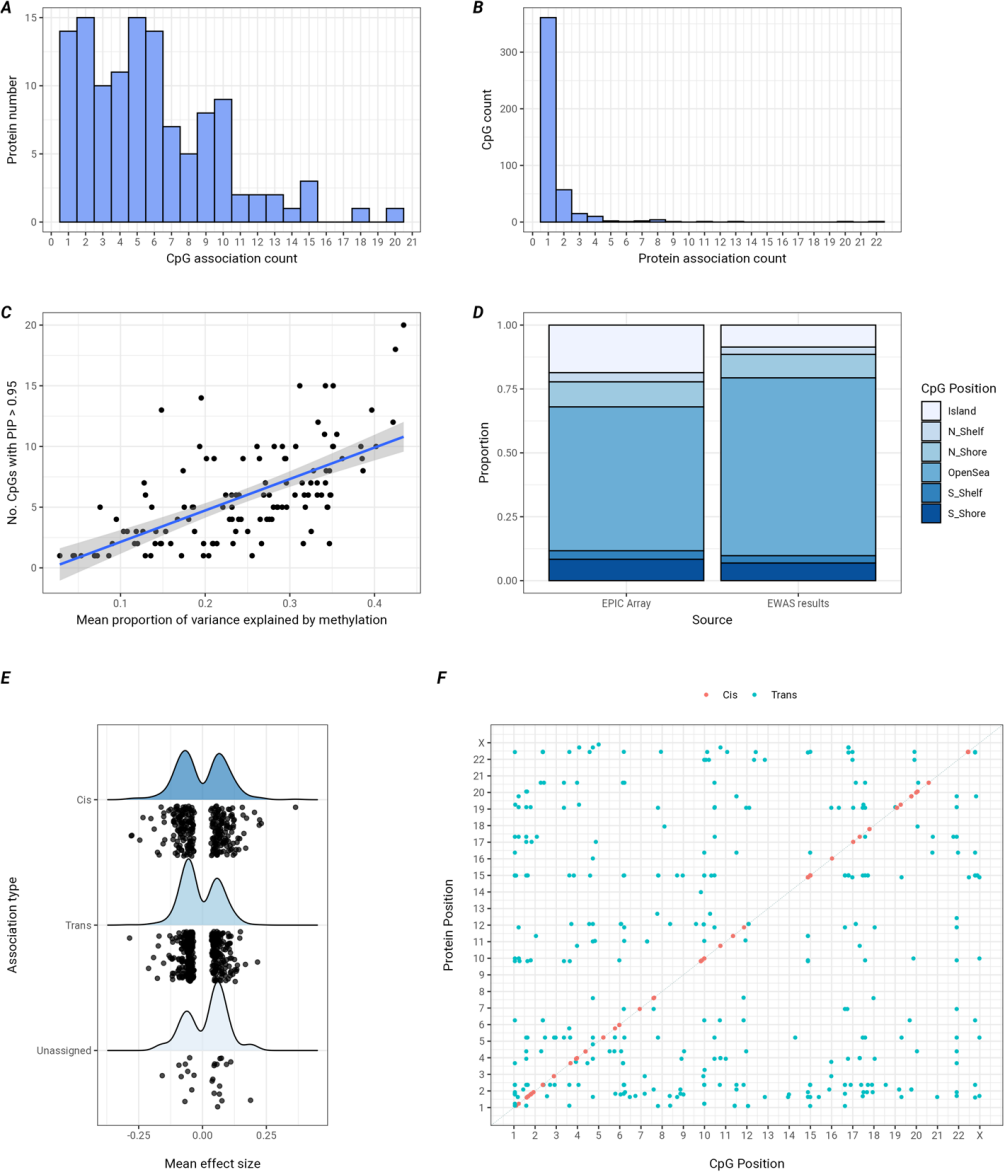
Summary of 697 protein ~ CpG associations from the Bayesian EWAS results. **A** The distribution of number of proteins by number of CpG associations; **B** The distribution of number CpGs by number of protein associations; **C** The correlation between the number of CpG association of each protein, by the mean proportion of variance explained by all CpG loci; **D** The proportion of CpGs in regions, specified by relation to CpG islands for the EPIC array and for the Bayesian EWAS results, demonstrating enriched results in Open Sea and reduced in Island regions; **E** Mean effect size of associations by association type, demonstrating the effect size is similar whether the association is in cis or trans. Unassigned associations are those for which the protein gene could not be annotated to a position in GRCh37 (*N* = 24, Additional file 3: Methods M3); **F** Each association plotted by genomic position of the protein gene and CpG probe demonstrating the distribution of associations across the genome

The distribution of associations by protein and CpG for the 697 lead findings from the Bayesian EWAS are displayed in Fig. 2. These demonstrate that most proteins (59%) have 6 or fewer associations, with a maximum number of 20 for P02750 (Leucine-rich alpha-2-glycoprotein). There was a strong concordance (*r* = 0.64) between the number of CpGs with PIPs >0.95 and the mean proportion of variance explained by all CpG loci for each protein, supporting the investigation of DNAm proxies for proteins (Fig. 2C). Most CpGs (79%) had one protein association, with up to 22 associations for one CpG (cg06072257, in open sea on chromosome 1, see Additional file 1: Table S5). Correlation analysis of the 22 associated proteins revealed Pearson r values between − 0.37 and 0.62 between pairs of proteins, with several demonstrating no intercorrelation (Additional file 1: Table S6). Network analysis with StringDB [19] revealed both functionally related and unrelated proteins, including those involved in the complement and coagulation cascades. This locus was associated with 11 different immunoglobulin components, 3 complement proteins, in addition to Ficolin-3, Angiotensinogen, Serotransferrin, Alpha-1-acidglycoprotein 1, Cell division cycle 5-like protein, C4b-binding protein alpha and beta chains and Vitronectin (Additional file 2: Figs. S1. and S2.). Across the 133 Bayesian protein EWASs, the significant loci were not distributed proportionally across the genomic regions (e.g. OpenSea, CpG Islands etc.) captured by the array (X^2^: 62.99, df = 5, *P* = 2.92 × 10^− 12^, Fig. 2D). For example, there was an enrichment of findings within OpenSea regions but fewer findings than would be expected in CpG Islands. The majority (56%) of the effect sizes were identified in *trans* locations although there were no differences by direction or magnitude between *cis* and *trans* associations (Fig. 2E). The distribution of *cis* and *trans* associations across the genome is displayed in Fig. 2F.

### EWAS catalogue

The EWAS catalogue [20] was searched (download date: 05/08/25) to identify any previously reported associations for our 697 results from the Bayesian model. After filtering to entries from “whole blood” and *P* < 3.6 × 10^− 8^ and matching on UniProt ID, the protein gene or the word “protein”, four previously identified associations were identified - all from our previous EWAS of SomaScan proteins [21] (Additional file 1: Table S7). That study (n_individuals_ = 774, n_proteins_ = 4,058) [21] featured 60 unique proteins that were also present in the MS dataset. There were 12 CpG loci with *P* < 3.6 × 10^− 8^ for 8 of these 60 proteins, of which four (associating with three proteins) had a PIP >0.95, all with concordant effect size directions in our current analyses. If the P-value threshold is relaxed to < 0.05, there are 4107 CpG loci demonstrating associations with the 60 proteins, of which 24 had a PIP >0.95 in the current study, with a correlation of effect sizes of 0.8 (*P* = 2.6 x 10^-6^). It is important to note the differences between the methods employed by the two studies (marginal regression vs. joint and conditional modelling of the CpGs). Further, the SomaScan EWAS considered plasma proteins rather than serum as in the current MS proteome analysis. Although both studies utilised data from Generation Scotland, the biosamples were taken at different time points, with the blood samples for plasma being obtained between 2015 and 2018, compared to between 2006 and 2011 for serum samples analysed via MS [22].

To identify any traits previously associated with the 457 lead loci, we again filtered to entries from “whole blood” with *P* < 3.6 × 10^− 8^ and further to studies with *N* >1000. 231 of the 457 CpGs have previous trait-associations documented in the EWAS catalogue, 105 of these with more than one trait (see Additional file 1: Table S8). This demonstrates that our results align with and expand on previous associations identified in other EWASs. For example, we identified additional and relevant protein associations of cg00574958 (*CPT1A* gene) and cg06500161 (*ABCG1* gene), previously associated with multiple metabolic traits [23, 24]. Similarly, we identified relevant protein associations for cg19693031 (*TXNIP* gene) previously associated with type 2 diabetes [23, 25] and cg07839457 (*NLRC5* gene) previously associated with immune-related proteins such as CD48 antigen [26] or traits such as rheumatoid arthritis [23].

### EpiScores

We generated 133 protein EpiScores using the Bayesian GMRMomi approach which, through joint and conditional modelling of all CpG loci provides a parsimonious solution for each protein. The number of CpGs with non-zero weights for each EpiScore, the majority of which (91%) can also be found on the EPICv2 array, are summarised in Additional file 1: Table S9.

**Fig. 3:**
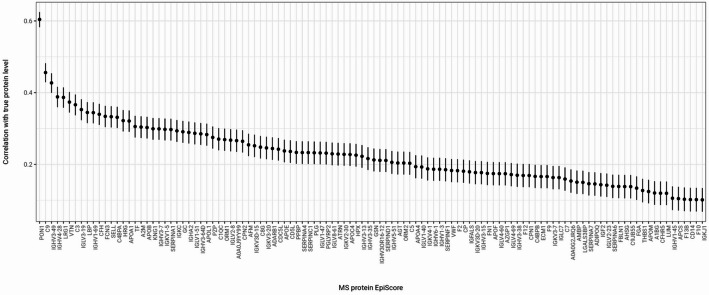
Pearson correlation of 112 EpiScores and proteins in the Generation Scotland test set. Test set N = 3,463. Correlation results displayed for 112 EpiScores where Pearson *r*>0.1 and *P* < 0.05 using the EPICv1 loci. Central dot represents Pearson r and the error bars represent 95% confidence intervals. Proteins are labelled by gene, except for Ig-like domain-containing protein 1 (A0A0G2JRQ6) and 2 (A0A0J9YY99), annotated by UniProtID. These proteins were annotated to scaffolds or patches in build hg19 and have not been assigned gene names (see Additional file 3: Methods M3.). Transferrin (C9JB55, 75 amino acids) is also labelled by UniProtID as it originates from the same gene as Serotransferrin (P02787, 698 amino acids, labelled TF)

Of the 133 Protein EpiScores, 112 had Pearson *r* > 0.1 and *P* < 0.05 with rank-based inverse normalised proteins when projected into an independent Generation Scotland test set (*n* = 3,463) (Fig. 3., Additional file 1: Table S10). These patterns largely persisted when we reprojected the EpiScores using loci common to both EPICv1 and EPICv2 arrays (Additional file 1: Table S11, Additional file 2: Fig. S3).

**Fig. 4:**
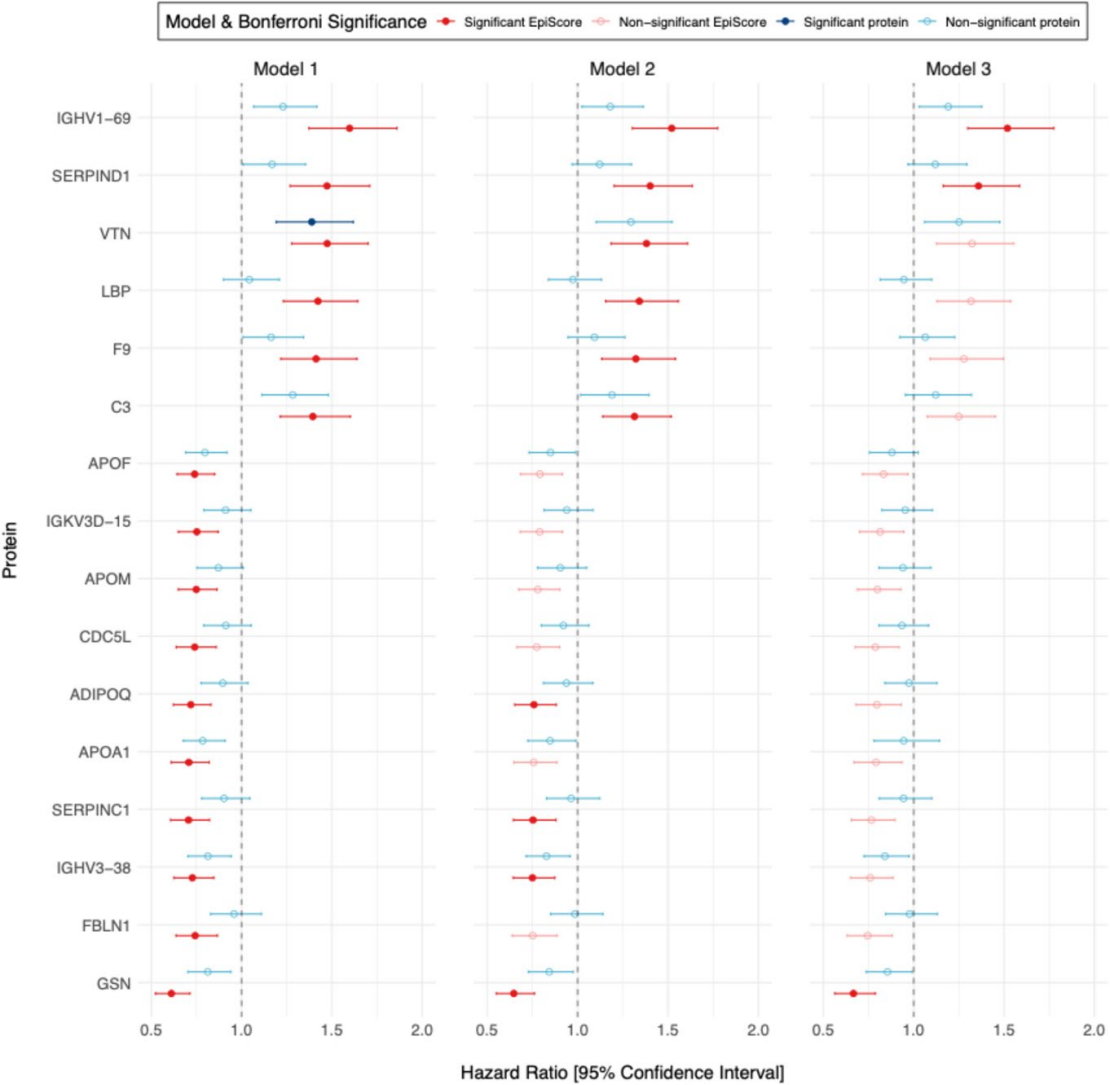
EpiScore and measured protein hazard ratios for time-to incident cardiovascular disease. Results are displayed where either protein or EpiScore demonstrate Bonferroni-significant associations (*P* < 0.05/112) in model 1. Model 1: TTE ~ EpiScore/Protein + age + sex; Model 2: TTE ~ EpiScore/Protein + age + sex + BMI + smoking + alcohol; Model 3: TTE ~ EpiScore/Protein + age + sex + BMI + smoking + alcohol + diabetes + hypertension + HDL cholesterol + Total cholesterol + average systolic blood pressure + average diastolic blood pressure. EpiScore/Protein denotes EpiScore or protein as a predictor variable. HR = Hazard Ratio per SD of the predictor, CI = 95% confidence interval. Colour in bold denotes significance at *P*_Bonferroni_ < 4.46 × 10^− 4^ (= 0.05/112). Proteins are labelled by gene, with the exception of Ig-like domain-containing protein 1 (A0A0G2JRQ6) and 2 (A0A0J9YY99), annotated by UniProtID, which were annotated to scaffolds or patches in build hg19 and have not been assigned gene names (see Additional file 3: Methods M3.). Transferrin (C9JB55, 75 amino acids) is also labelled by UniProtID as it originates from the same gene as Serotransferrin (P02787, 698 amino acids, labelled TF)

The 112 EpiScores and their corresponding proteins were then studied in relation to incident cardiovascular disease via Cox proportional hazards models with a follow-up duration of up to 17.6 years (*n* = 3,345, after excluding those with a prevalent cardiovascular disease diagnosis – see Methods) (Fig. 4).

We compared the number and magnitude of statistically significant associations (hazard ratios) for both the protein EpiScores and directly measured proteins in relation to incident cardiovascular disease. After adjustment for age and sex, 16 protein EpiScores demonstrated a significant relationship with the composite cardiovascular disease outcome, compared to only one of the measured proteins (P_Bonferroni_ < 0.05/112 = 4.46 × 10^− 4^); 52 EpiScores and 21 proteins – mapping to 56 unique proteins – were significant at a nominal *P* < 0.05 threshold (Additional file 1: Tables S12 and S13). Furthermore, the absolute values of the log-hazards (per standard deviation of the predictor variable) were consistently greater for the EpiScores compared to the corresponding measured proteins (Fig. 4., full results: Additional file 1: Table S13 and Additional file 2: Fig. S4). These findings remain nominally significant (*P* < 0.05) upon further adjustment for covariates relevant to cardiovascular disease. The proportional hazards assumption was met for all models (Schoenfeld residual P_global_>0.05, P_EpiScore/Protein_ >0.05, Additional file 1: Tables S14 & S15) with the exception of one EpiScore (Cell division cycle 5-like protein, CDC5L, Q99459) and one protein (Vitronectin, VTN, P04004).

Finally, we compared nested Cox proportional hazard models to determine if the EpiScore augmented the measured protein in the incident cardiovascular disease analyses. Here, we considered the 17 instances where both the model with the EpiScore and the model with the corresponding, directly measured protein had nominally significant (*P* < 0.05) associations for the EpiScore and protein. Adding the EpiScore to a model controlling for age, sex and the measured protein resulted in a significantly improved model fit for 10 of the 17 models at *P* < 0.003 (0.05/17), (Additional file 2: Fig. S5., and Additional file 1: Tables S16 and S17).

## Discussion

Here, we present the first large scale epigenome-wide assessment of the highly abundant serum proteome, as measured by mass spectrometry. Using two different regression frameworks, we revealed 15,855 significant associations via a marginal linear approach and 697 associations using a penalised Bayesian approach. 505 of the protein ~ CpG associations overlapped between the two methods. The two EWAS approaches offer different insights into the relationship between the methylome and the proteome. As the marginal regression models consider each CpG in isolation, ignoring any correlation patterns across the genome, it identifies a large number of associations. By contrast, the penalised Bayesian approach implicitly performs fine mapping through the joint and conditional analysis of all CpGs. This yields parsimonious solutions for downstream analyses and EpiScore applications [16].

As we have shown for other complex traits, such as markers of metabolic health [27], the method used to conduct an EWAS has major implications on the number of significant loci identified. Here, a marginal approach identified 15,855 lead loci, compared to 697 in the joint and conditional penalised Bayesian regression. Both methods offer valid insights. The former highlights associations for intercorrelated CpGs, potentially across genes which are functionally related. The latter identifies a parsimonious set of high-confidence lead loci, reducing the chance of false-positive findings.

Focussing on the Bayesian EWAS, our results include both previously unreported associations and those that align with and expand upon results reported in the literature. For example, some of our loci, mapping to *ABCG1*,* CPT1A* and *TXNIP*, have been associated with lipid and metabolic traits such as triglycerides, BMI, and type 2 diabetes [23–25, 28, 29]. Four CpGs within *ABCG1*, which encodes a protein involved in cholesterol transport [30], associated with 12 different proteins. For example, cg06500161 had 8 *trans* associations with apolipoprotein F, apolipoprotein C4, afamin, gelsolin, vitronectin, apolipoprotein A-I, antithrombin-III, and plasminogen. Additionally, cg00574958 (*CPT1A*, important for mitochondrial oxidation of long-chain fatty acids [31]), was found here to be associated with Apolipoprotein E, Apolipoprotein B-100, and two immunoglobulin components, complementing previous protein associations with APOC3 and CRP [21, 32]. We identified associations for cg19693031 (*TXNIP*, an oxidative stress mediator, particularly associated with diabetes [33]) and eight different proteins, including those involved in the innate immune response such as Ficolin 3, Attractin and Complement component C9, which provides further insight into the link between diabetes and inflammation [34]. Finally, we build on previously identified associations between CpGs annotated to *NLRC5* and immune-system proteins [26]. We found associations between cg0783945 (*NLRC5*) and seven proteins, including four immunoglobulin components, 2 complement proteins and plasminogen whilst cg16411857 (*NLRC5*) was also associated with an immunoglobulin component, thus reinforcing the role of the *NLRC5* as a regulator of immune responses [35].

In our results, the most pleiotropic CpG site (cg06072257) associated with 22 proteins. The CpG itself is not directly annotated to a gene, but is situated near *UBIAD1*, which encodes UbiA Prenyltransferase Domain Containing 1, a protein involved with antioxidant processes [36]. It has previously been associated with prevalent breast cancer [23], incident COPD [23], an age-by-sex interaction [37] and the levels of 17 proteins including CRP, SERPING1, CHAD and CGA [21, 32]. However, none of these proteins previously identified were assessed in our dataset. A network analysis using StringDB [19] highlighted multiple interactions (Additional file 2: Fig. S2). For example, complement 8 alpha and gamma chains and vitronectin are classified as having ‘known interactions’ (KEGG annotated pathway: Complement and coagulation cascades, via StringDB). However, in our data, vitronectin did not correlate strongly with the complement 8 alpha or gamma chains (r_Pearson_ = −0.07, 0.017, respectively) and only shared this one high confidence CpG locus. Further, Ficolin-3 and complement factor H, which were weakly correlated (r_Pearson_ = 0.16, S17), are both annotated to the serine-type endopeptidase complex (Protein complexes annotated by the Gene Ontology Consortium, as of August 2022, via StringDB). The association of this CpG locus with proteins of related functions, in our results and previous EWASs [21] suggest it could play a regulatory role or reflect changes in the activity of these pathways. However, this analysis was partly limited by incomplete recognition of genes for immunoglobulin proteins in the StringDB database and further experimental evidence is required to explore this in detail.

In independent train/test Generation Scotland subsets, we generated statistically significant EpiScores for 112 proteins using CpGs from the EPICv1 array. As suggested from our sensitivity analyses, not all will translate perfectly to different Illumina array versions. Sixteen of the generated EpiScores were associated significantly with incident cardiovascular disease compared to just one measured protein, in an age and sex adjusted model. In general, the hazard ratio estimates for EpiScores were either higher (where HR > 1) or lower (where HR < 1) than the respective measured protein estimates. Further, it is not necessarily the EpiScores which correlate the highest with their respective protein which demonstrate the strongest disease associations. For example, Gelsolin (GSN, P06396) associates with cardiovascular disease with a hazard ratio of 0.6, *P* = 4.9 × 10^-10^ and demonstrates a Pearson r of 0.21 with its paired measured protein. This pattern extends to models adjusted for cardiovascular-relevant covariates, where three EpiScore associations remained Bonferroni significant.

Many of the EpiScore findings align with previously studies on proteins and cardiovascular disease. For example, vitronectin (P04004) has already attracted considerable interest as a putative biomarker for cardiovascular disease. It is a glycoprotein found both in blood, alpha granules of platelets and the extracellular matrix, with suspected roles in platelet aggregation following vascular injury and, via plasminogen activator inhibitor-1, a role in reducing thrombus clearance [38]. Vitronectin levels have been shown to correlate with the extent of coronary atherosclerosis [39], to be higher in patients with acute coronary syndromes [40] and be an independent risk factor for adverse cardiovascular events in patients undergoing percutaneous cardiac interventions [41]. Thus, it has been suggested as a biomarker to increase the accuracy of acute coronary syndrome diagnosis [42]. Our results (P04004_EpiScore_ HR per SD (95% CI): 1.48 (1.28,1.7), compared to P04004_measured_ 1.39 (1.19, 1.62)), which focused on longer term cardiovascular disease prediction, suggest it would be worth considering an EpiScore for vitronectin alongside the measured protein itself when investigating biomarker utility. Similarly, lipopolysaccharide-binding protein (P18428) has also been associated with increased risk of cardiovascular disease [43], and the EpiScore demonstrated a stronger association with incident cardiovascular disease 1.42 (1.23–1.64) compared to 1.04 (0.9–1.2) for the measured protein.

By contrast, Heparin cofactor 2 (P05546, SERPIND1) is a thrombin inhibitor, and has predominantly been suggested to be protective against cardiovascular disease [44, 45]. However, our findings showed an increased risk of a cardiovascular diagnosis or death with increasing concentrations (EpiScore HR 1.47 [1.27–1.71], measured protein 1.17 [1.01–1.35]).

Gelsolin (P06396, GSN) is another interesting example. This protein facilitates actin filament recombination and circulating GSN is proposed to mitigate the development of atherosclerosis through multiple pathways including inflammatory cell migration and interleukin release and limiting endothelial injury [46]. In our results, the GSN EpiScore demonstrated a significant protective association with incident cardiovascular disease (HR: 0.61 [0.52,0.71], *P* = 4.96 × 10^− 10^), whilst the measured protein demonstrated a protective, but non-significant relationship after correction for multiple comparisons (HR: 0.81, *p* = 0.005).

Blood-based DNAm measures methylation predominantly in leucocytes [47]. High abundant proteins, comprising up to 99% of plasma proteins by mass [12] are mostly synthesised in the liver and secreted into plasma [48]. Therefore, the association between blood-based DNAm measurements and protein abundance is indirect. Nevertheless, we have demonstrated that blood-based DNAm significantly associates with relative protein abundance in 112 of 133 proteins assessed. We hypothesise that DNAm patterns reflect biological changes underpinning variation in these proteins, largely influenced by factors impacting protein regulation and synthesis such as inflammatory and metabolic states.

For example, ApoA-1 is a major component of high-density lipoproteins, transporting cholesterol from tissues to the liver for excretion, with additional immuno-modulatory and anti-inflammatory roles [49]. Synthesis of ApoA-1 occurs principally in the liver and also the intestine, regulated by hormonal mediators such as oestrogen, thyroid hormone and insulin [50], in turn impacted via lifestyle factors such as diet and exercise [51]. Further, systemic inflammation, a feature of metabolic syndrome, can inhibit Apo-AI production via inflammatory cytokines such as IL-6 and TNF-alpha [52]. There is a well-established impact of inflammation and metabolic state on DNA methylation in blood [9, 32] and thus it is likely these common features underpin correlations between EpiScores and proteins. Multiple studies have identified an association of ApoA-1 or ApoA-1:lipid ratio and cardiovascular disease [53, 54]. We found Apolipoprotein A1 (ApoA-1) was associated with decreased hazards of cardiovascular disease (HR_measured protein_:0.79, *P* = 0.001, HR_EpiScore_: 0.71, *P* = 4.76 × 10^− 06^) in the age and sex adjusted model.

Though the strength of the mass spectrometry approach is its untargeted nature, reducing potential selection bias, it does not easily measure low abundance proteins. As approximately 20 proteins constitute 98% of the protein mass in human plasma, mass spectrometry may lose valuable information from low-abundance proteins which remain highly relevant for health and disease, unless particular strategies are employed to allow their accurate detection [55]. An example of this could be interleukins, which occur at concentrations of ng/L in contrast to, for instance, apolipoproteins at concentrations of g/L [56].

Alternative mass spectrometry approaches such as the Seer platform [57] have also been used to develop protein EpiScores (termed epigenetic biomarker proxies). As further test-sets become available, it will be interesting to compare the performance of EpiScores for the same protein generated on different platforms.

As the Generation Scotland cohort is predominantly of white European ancestry and limited to those living in Scotland, these results are not necessarily generalisable to other populations, although previous work has demonstrated that EpiScores for diabetes risk, metabolic traits, CRP and smoking have all translated well to other cohorts, including those of diverse ancestries [11, 27, 32, 58]. As the methylation and protein data are cross-sectional, results and interpretation could be strengthened by analysis in longitudinal datasets, allowing within subject comparison of changes in methylation and protein levels. Additionally, DNAm is only one form of epigenetic regulation. To form a complete understanding of the relationship between the epigenome and the circulating proteome we could also consider additional factors such as histone modification, chemical modification of RNA and mitochondrial gene expression [7].

Our analyses demonstrates that protein EpiScores exhibit significant relationships with incident cardiovascular disease where measured proteins do not and tend to demonstrate stronger hazard ratio point estimates. However, further modelling, in a larger dataset, including other known cardiovascular disease risk factors should be undertaken to probe this relationship further.

## Conclusions

Here, we conducted the first large scale methylome-wide analyses of the high abundant serum proteome as measured by mass spectrometry. Using two separate statistical frameworks, we identified 505 common CpG-protein associations, the majority of which were *trans* associations. We find a complex interplay between these omics layers, including a single CpG (cg06072257) associating as a *trans* locus with 22 proteins, whilst most CpGs are associated with a small number of proteins. Furthermore, we generated EpiScores for 112 proteins, 16 of which demonstrated both significant and stronger associations with incident cardiovascular disease compared to the respective measured proteins. There was also evidence for additive effects when including both the measured protein and its corresponding EpiScore in the same model.

Our results demonstrate the potential for protein EpiScores as disease biomarkers, particularly applicable to non-communicable diseases associated with environmental and lifestyle factors. The potential for applications in disease prediction, evaluating therapeutic intervention, risk stratification and precision medicine warrant further investigation alongside proteome measurements.

## Methods

### Generation Scotland

Generation Scotland is an epidemiological study with comprehensive DNA, clinical, and socio-demographic data from approximately 24,000 volunteers with linkage to medical records [59]. Participants were recruited from across Scotland, aged 17–99, between 2006 and 2011. Blood samples were taken during the initial clinic visit for just over 20,000 volunteers, alongside health, cognitive and lifestyle questionnaires. Participants provided informed consent to electronic-health records linkage to both secondary and primary care data, allowing analysis of prevalent and incident disease.

### Mass spectrometry proteomics

Measurement of the circulating proteome in Generation Scotland was carried out using a high flow-rate liquid chromatography tandem mass spectrometry, using SWATH acquisition [60]. Data processing was performed with DIA-NN, using a spectral library approach [61].This generated data for 439 inferred proteins, for 15,818 participants at the time of analysis.

Full details of the protocol have been described previously [62]. In brief, serum samples were pre-processed for protein denaturation and trypsinisation, prior to liquid chromatography - mass spectrometry (LC)-MS, using the Agilent 1290 Infinity II system and TripleTOF 6600 mass spectrometer (SCIEX) and a scanning SWATH method [60]. Output data were processed by DIA-NN [61], identified using a spectral library [63] with precursor false discovery rate (FDR) set to 1%. R was used for further post-processing including within-batch drift correction using a previously described method [64] and between-batch correction, using the “limma” v3.54.2 algorithm [65]. Identified signals were mapped to Universal Protein Resource (UniProt) IDs [66]. 133 of the signals were uniquely mapped to one protein (Additional file 1: Table S18) and the remaining 306 were mapped to multiple possible target outcomes (Additional file 3: Methods M1). For computational efficiency, we focus here on the 133 individual proteins, the values for which were rank-based inverse normalised before being taken forward for further analysis.

### DNA methylation

Whole blood-based DNAm measurements were profiled on samples from the Generation Scotland baseline appointment, on sodium bisulphite treated DNA, with the Illumina Infinium HumanMethylationEPIC BeadChip array v1.0 [67]. Sample processing and quality control (QC) of the methylation data is described in Additional file 3: Methods M2, and has previously been described in full [22]. Briefly, DNAm was profiled in four separate sets (Post QC: N_Set1_ = 5,087, N_Set2_ = 459, N_Set3_ = 4,450, N_Set4_ = 8,873, Total *N* = 18,869) [22]. Samples were removed if the median methylated signal intensity was over three standard deviations lower than the expected value, where the methylation-derived sex differed from self-reported sex and if >0.5% CpGs in the sample had a detection *P*-value >0.01 (or >1% of CpGs with a detection *P*-value >0.05 for set 1). Poorly performing probes were also removed if the beadcount was < 3 in >5% of samples or >1% of the samples had a detection *P*-value of >0.01 (>0.5% samples with detection *P*-value >0.05 in set 1). A total of 752,722 CpG sites were included following quality control. Normalised methylation M-values were used for downstream analyses. 14,671 individuals from the Generation Scotland cohort had complete methylation and protein data for analysis.

### Epigenome-wide association studies (EWASs)

EWASs were performed to identify CpG-protein associations. We first employed a marginal linear model approach (separate linear model for each possible CpG-protein association), using OmicS-data-based Complex trait Analysis (OSCA, v0.46.1) [15]. Marginal regression analyses do not account for the correlation structure between CpGs across the genome, considering each locus in isolation. This approach is most commonly taken in omics association studies but can lead to issues with genomic inflation [68] which was observed here. We subsequently employed a newly developed Bayesian approach (GMRMomi) which models all CpGs jointly and conditionally on each other for each protein [16]. This estimates CpG effects whilst considering relationships between probes, selecting highly influential probes amongst those which are correlated. Therefore, whilst the marginal linear analysis seeks to identify all CpGs associated with the phenotype of interest, the penalised Bayesian approach facilitates both dimensionality reduction and fine mapping to identify the most strongly influential probes and parsimonious EpiScore signatures. In order to reduce the computational burden of the >400 million linear and 133 Bayesian EWASs, methylation M-values were pre-regressed for age, sex and measurement batch using the limma package (version 3.60.4) in R [65]. Residuals from the output of a linear model for each CpG were scaled to have a mean of zero and unit variance prior to the EWASs. Demographics for the 14,671 included individuals can be found in Additional file 1: Table S19. All analyses outside of OSCA and GMRMomi were conducted in R version 4.4.1.

### OSCA linear

Rank-based inverse normalised values for 133 proteins were taken forward to mixed-effects linear regression analyses that adjusted for age and sex as fixed effects and a kinship matrix as a random effect using the lmekin function (coxme package, version 2.2.20 [69]). The kinship matrix accounts for relatedness within the known family structures in Generation Scotland. Residuals from each model (one per protein) were then scaled to have mean of zero and unit variance prior to downstream analysis.

The fast-linear option within OSCA was used for the frequentist EWASs, with an iterative approach for including covariates known to impact methylation [70], see Fig. 1. Added covariates included: estimated white blood cell proportions (Houseman method [71], with neutrophils dropped to minimise collinearity) in model 2. In model 3, we further included body mass index (BMI, kg/m^2^) and smoking pack-years, where one smoking pack-year equates to smoking 20 cigarettes per day for one year, both known to affect DNA methylation [27, 58]. Both variables were log transformed (a constant of 1 was added to all values in the smoking variable to account for never smokers in the transformation), to minimise skew in the data. Missing data (BMI: *n* = 92, smoking pack-years, *n* = 275) were mean-imputed using the impute_mean function from the missMethods package, version 0.4.0 in R [72], prior to log transformations. Finally, in model 4, we added the first 20 principal components of the methylation data to account for possible unmeasured confounding, given ongoing evidence of model inflation (Additional file 2: Fig. S6) and as previously employed in EWASs in GS [22, 27]. The relationship between the principal components and other continuous covariates is illustrated in Additional file 2: Fig. S7. The strongest correlation observed were those between PC6 and eosinophil proportion and PC9 and eosinophil proportions (Pearson *r* = 0.25 and *r* = −0.25, respectively, Additional file 1: Table S20). The strongest correlations observed between proteins and PCs were 0.09 (PC14 and C3, P01024) and − 0.09 (PC15 and PON1, P27169), see Additional file 1: Table S21. A Bonferroni-corrected threshold of P_Bonferroni_ < 2.71 × 10^− 10^ was set for statistical significance. This was calculated using *P* < 3.6 × 10^− 8^ as the epigenome-wide significance threshold divided by the number of proteins assessed (133) [18].

### GMRMomi

GMRMomi is a software implementation of Bayesian penalised regression [16, 17] based upon a framework proposed for genomics data (GMRM), adapted for large-scale multi-omics data. The method utilises Gibbs sampling to generate draws from the posterior distribution, considering the underlying genetic architecture and intercorrelation of CpG sites, modelling all CpGs jointly and conditionally on each other. In addition to controlling for known covariates, this method implicitly controls for unknown variables such as white-cell proportions, which would usually be estimated from the methylation data itself. This contrasts with the marginal analysis undertaken by OSCA, which considers each probe separately.

Methylation M-values were prepared as above. Rank-based inverse normally transformed protein levels were regressed on age, sex, logarithmic transformation of BMI, and the logarithmic transformation of smoking pack-years (+ 1). Any missing data (BMI: *n* = 92, smoking pack-years: *n* = 275) were mean imputed. The residuals from these linear models were scaled (mean zero, unit variance) and taken forward for further analyses. Prior mixture variance proportions were set to 0.0, 0.001, 0.01, and 0.1, equivalent to negligible, small, medium and large CpG effect sizes, as previously used for Bayesian EWAS studies of the circulating proteome [73]. 2000 model iterations were run for each protein, with 750 ‘burn-in’ iterations discarded prior to averaging the effect sizes over the last 1250 posterior samples. A posterior-inclusion probability (PIP) of >0.95 was used to select robust CpG-protein associations (Fig. 1). The mean proportion of variance explained by all CpG loci for each protein was calculated as the mean variance explained by methylation probes divided by total variance across the last 1250 iterations.

### Annotation of proteins and methylation sites

CpG sites were annotated using the minfi package in R (*IlluminaHumanMethylationEPICanno.ilm10b4.hg19)*, version 1.50.0 [74], to establish chromosome, probe position, relation to CpG-island and any nearby genes. Protein gene annotations were performed in R using BioMaRT and Ensembl (Genome reference consortium build37, GRCh37, to establish chromosome and transcription start-site (TSS) [75, 76].

CpG sites were characterised as being in *cis* (within 1 Mb) or *trans (*outside of this region or on a different chromosome) of the TSS of the associated protein’s gene. It is important to note that these annotations are for positional and descriptive purposes, irrespective of the primary tissue responsible for protein synthesis and we make neither a directional nor causal assumption about the nature of the relationships between CpGs and proteins.

Three proteins could not be fully annotated to a genomic position in GRCh37, for further details see Additional file 3: Methods M3. A Chi-squared test and post hoc Z-tests assessed whether the distribution of CpG location (e.g., part of a CpG island) for the significant EWAS loci, differed from the distribution of all probes on the array.

Further analysis included an EWAS catalogue [20] search for previous associations for CpGs identified as statistically significant in the EWASs, along with the characterisation of pleiotropic loci and assessment for pathway enrichment in probe-associated genes (Additional file 3: Methods M4 & M5).

### Protein episcores

The 14,671 Generation Scotland participants were split into training and test datasets to build protein EpiScores. The training dataset contained 6,816 individuals from measurement Sets 1, 2 and 4. To minimise overfitting, all individuals (*n* = 3,671) within the same family pedigree [77] as participants in the test dataset were removed from the training dataset. The test dataset contained 3,463 unrelated individuals who had DNAm processed together in Set 3. Demographics for the training and test sets can be found in Additional file 1: Tables S22 & S23. In the training dataset, GMRMomi was run as previously specified (prior mixture variance proportions were set to 0.0, 0.001, 0.01, and 0.1, total iterations to 2000 and 750 burn-in iterations. The mean posterior CpG weights, calculated from post-burn-in iterations, for each of the 133 protein regression models were extracted. EpiScores were then projected into the test set to create protein EpiScores (additive sum of all CpG weights multiplied by the measured CpG M-values). We additionally calculated the proportion of CpG loci for each EpiScore which can be found on the EPICv2 array [78] (Additional file 1: Table S9) and re-calculated the protein EpiScores using only the loci on both the EPICv1 and EPICv2 arrays. To gauge the translatability of these EpiScores to data where methylation has been measured using the EPICv2 array we assessed the correlation between both sets of EpiScores and their paired measured protein (Fig. 3., Additional file 1: Table S11).

### Protein episcore versus measured proteins: associations with incident cardiovascular outcomes

EpiScores which demonstrated a significant correlation with the measured protein level (Pearson *r* >0.1 and uncorrected *P* < 0.05), were taken forward for further analysis in the test dataset (*N*_individuals_ = 3,463). We explored the association of the protein EpiScores with incident cardiovascular disease using Cox proportional-hazards models [79]. Incident cardiovascular disease was defined as a composite outcome, including a diagnosis of coronary heart disease, ischaemic stroke, myocardial infarction and any death related to cardiovascular disease. Diagnoses were determined from secondary care records, using CALIBER/HDRUK consensus definitions [80] and cardiovascular disease related death using ICD codes I00-99, aligning with previous work on cardiovascular disease carried out in Generation Scotland [81] (for further details see Additional file 3: Methods M6). The censor date was set to the most recent date of linkage to disease data (August 2023) or non-CVD-related death, with a total follow up period of up to 17.6 years from baseline appointment (*N*_CVD−diagnoses and deaths_ = 191, *N*_censored_ = 3154). Any individuals with a diagnosis of cardiovascular disease received prior to their baseline appointment, were excluded from the analysis (*N*_prevalent_ = 116). For baseline demographic and outcome details see Additional file 1: Table S24.

Within the Generation Scotland test dataset, Cox proportional hazards models were run for each protein EpiScore and each mass spectrometry measured protein level and time-to incident cardiovascular disease using the survival R package, version 3.7.0 [82]. To compare the strength of association of both EpiScores and their paired, measured proteins with incident cardiovascular disease we ran models adjusting for age and sex. This approach has also been taken with other work assessing the potential of epigenetic biomarker proxies [57]. We additionally ran sensitivity analyses to control for further covariates: firstly BMI, smoking pack-years and alcohol consumption (units per week) (model 2) and finally also adding prevalent diabetes, prevalent hypertension, HDL cholesterol, Total cholesterol and average systolic and diastolic blood pressures (model 3). Missing data (BMI = 16, smoking pack-years = 4, alcohol units/week = 254, average systolic and diastolic blood pressures = 3, HDL cholesterol = 25 and total cholesterol = 21) were imputed using kNN (VIM v.6.6.2 [83]) For further information on the derivation of these variables see Additional file 3: Methods M6 and Additional file 1: Table S25 for ICD codes used. Both the EpiScores and proteins were rank inverse normalised prior to modelling. Finally, where both the EpiScore and measured protein were nominally significant (*P* < 0.05) in association with incident cardiovascular disease, nested models determined if the EpiScore improved fit by likelihood-ratio tests, (stats package in R, version 3.6.2) after adding it to a model adjusting for age, sex and the relevant (measured) protein.

## Supplementary Information

Supplementary Material 1.

Supplementary Material 2.

Supplementary Material 3.

## Data Availability

Applications for access to Generation Scotland data can be made to access@generationscotland.org. Further details can be found at https://genscot.ed.ac.uk/for-researchers/access. All code associated with this manuscript is available open access on GitHub under the GNU General Public License version 3.0 (https://github.com/marioni-group/MSprot_Epigenetics) and Zenodo [84]. Summary statistics are available in Zenodo under a Creative Commons Attribution 4.0 International license [85].
