# Supplementary material for: Methylome-wide association studies and epigenetic biomarker development for 133 mass spectrometry-assessed circulating proteins in 14,671 Generation Scotland participants
Source: Genome Biol. 2025 Dec 8;26:417. doi: 10.1186/s13059-025-03892-0 (PMC12683789; doi:10.1186/s13059-025-03892-0)
Supplement: Supplementary file 2 — Supplementary Material 2. [file 13059_2025_3892_MOESM2_ESM.docx]

**Supplementary Figures**


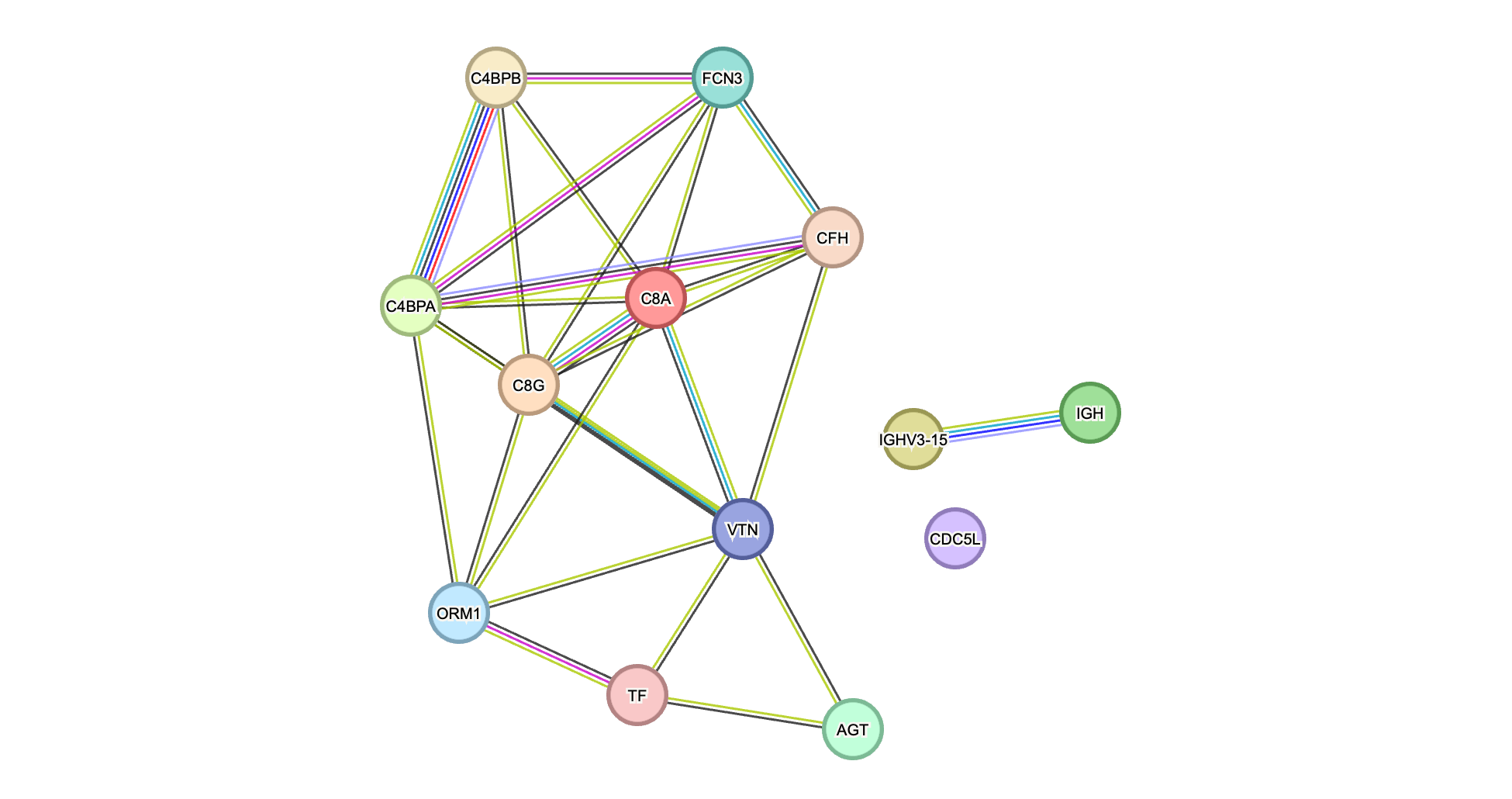


B

A


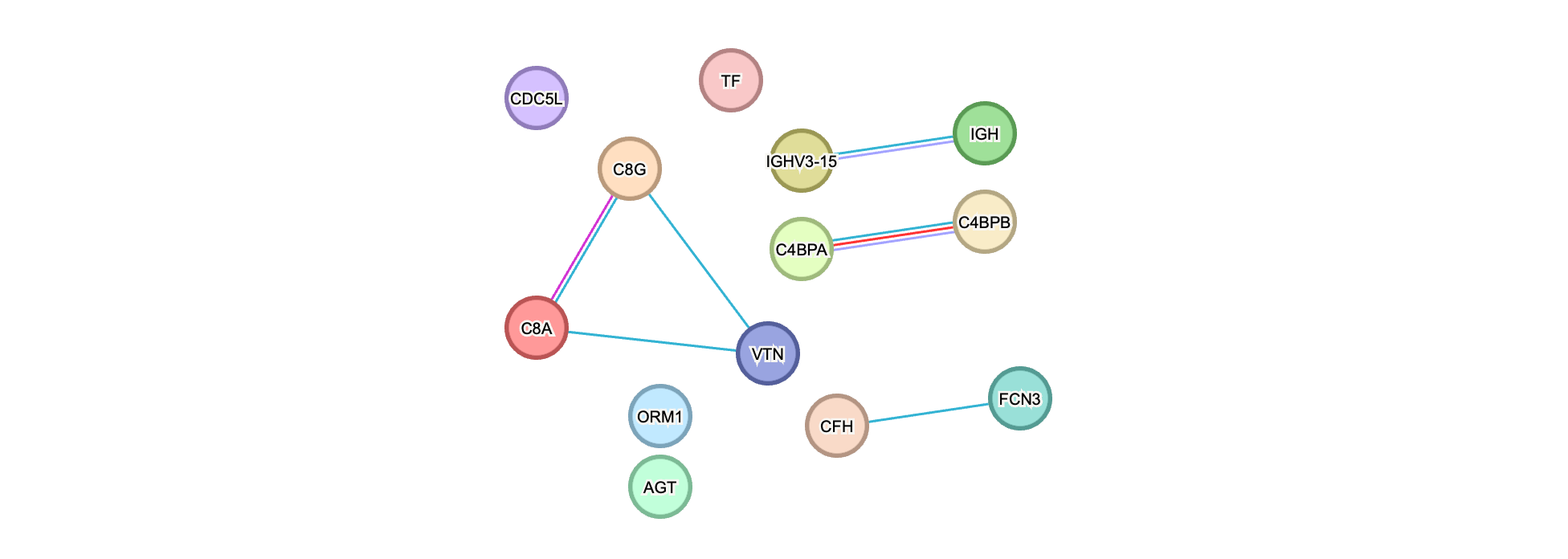


**Fig. S1. Network of identified protein genes from 22 proteins associated with cg06072257 from StringDB**^1^**.**

12 protein genes initially generated results in stringDB. By using the gene name “IGHV” for any immunoglobulin heavy variable which initially generated no results in StringDB, a further 4 proteins were assigned to this gene and included in the network. The remaining 6 proteins (all immunoglobulin components) could not be identified through the StringDB database (see supplementary table S19). **A:** reduced network demonstrating interactions based on experiments, databases, neighbourhood and gene fusion only. **B:** full network demonstrating interactions based on text-mining, experiments, databases, co-expression, neighbourhood, gene fusion and co-occurrence. See below for a direct excerpt from StringDB detailing protein descriptions and interaction type (screenshot from string-db.org, accessed 09/11/2024).


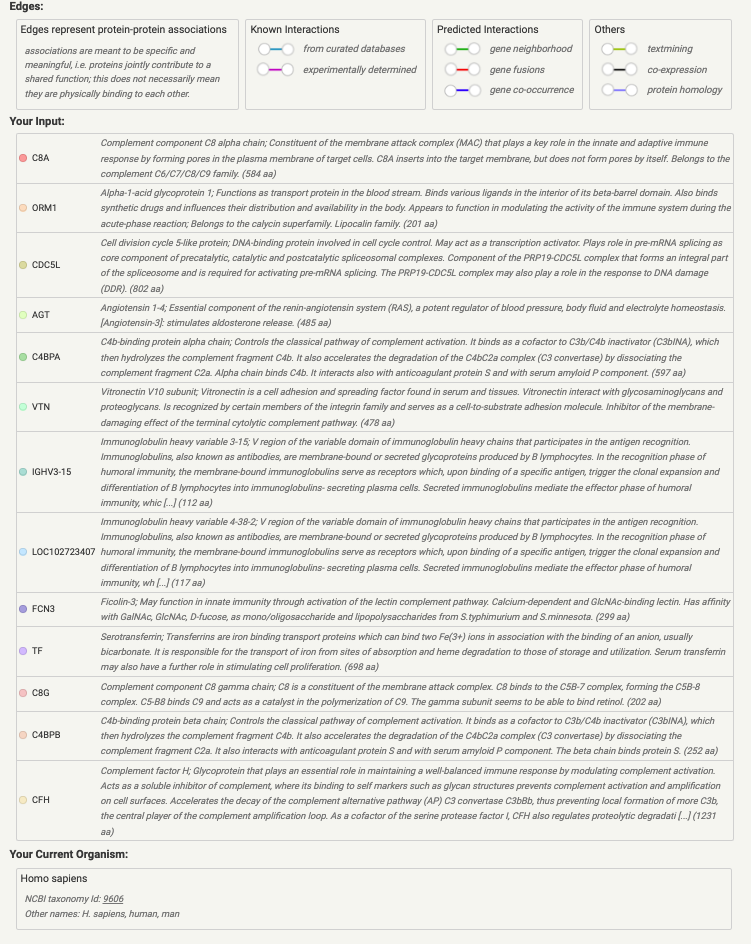


**Fig S2. Heatmap of proteins associated with cg06072257**.

Correlation values calculated by the rcorr function in the Hmisc package^2^ in R. Heatmap produced by ComplexHeatmap^3^ in R. Proteins are identified by their UniProt ID and name.

Ig kappa constant

Ig kappa variable 3-20

Cell division cycle 5-like protein

Ig lambda variable 8-61

Ig kappa variable 4-1

Probable non-functional Ig kappa variable 3-7

Ig heavy variable 3-7

Ig heavy variable 3-15

Probable non-functional Ig heavy variable 3-38

Ig heavy variable 6-1

Ig lambda variable 8-61

Ig heavy constant alpha 2

Serotransferrin

Angiotensinogen

Vitronectin

Alpha-1-acid glycoprotein 1

Ficolin-3

C4b-binding protein beta chain

C4b-binding protein alpha chain

Complement factor H

Complement component C8 gamma chain

Complement component C8 alpha chain

**Fig. S3. Correlations between EpiScores and their paired measured protein.**

Test set N = 3,463. Correlation results displayed for 112 EpiScores where Pearson R > 0.1 and P < 0.05 using the EPICv1 loci. 109 of the 112 EpiScores still correlated with R > 0.1 and P < 0.05 with their paired measured protein when generated using only the CpG loci on both the EPICv1 and EPICv2 arrays. Central dot represents Pearson r and the error bars represent 95% confidence intervals. Proteins are labelled by gene, except for Ig-like domain-containing protein 1(A0A0G2JRQ6) and 2 (A0A0J9YY99), annotated by UniProtID. These proteins were annotated to scaffolds or patches in build hg19 and have not been assigned gene names (see Supplementary Methods 3.). Transferrin (C9JB55, 75 amino acids) is also labelled by UniProtID as it originates from the same gene as Serotransferrin (P02787, 698 amino acids, labelled TF). CpG Loci refers to the loci include for EpiScore projection into the test set: EPICv1= all EpiScore weights were used. EPICv1&2 = the loci were subset to those common to both EPICv1 and EPICv2 arrays.

**Fig. S4. EpiScore and measured protein hazard ratios for time-to incident cardiovascular disease.** *Results are displayed where either protein or EpiScore demonstrate nominally-significant associations (P < 0.05) in model 1.* *Model 1: TTE ~ EpiScore/Protein + age + sex; Model 2: TTE ~ EpiScore/Protein + age + sex + BMI + smoking + alcohol, Model 3: TTE ~ EpiScore/Protein + age + sex + BMI + smoking + alcohol + diabetes + hypertension + HDL cholesterol + Total cholesterol + average systolic blood pressure + average diastolic blood pressure. EpiScore/Protein denotes EpiScore or protein as a predictor variable. HR = Hazard Ratio per SD of the predictor, CI = 95% confidence interval. Colour in bold denotes significance at P_Bonferroni_ < 4.46 x 10^-4^ (=0.05/112). Proteins are labelled by gene, with the exception of Ig-like domain-containing protein 1 (A0A0G2JRQ6) and 2 (A0A0J9YY99), annotated by UniProtID, which were annotated to scaffolds or patches in build hg19 and have not been assigned gene names (see Supplementary Methods 3.). Transferrin (C9JB55, 75 amino acids) is also labelled by UniProtID as it originates from the same gene as Serotransferrin (P02787, 698 amino acids, labelled TF).*

**Fig. S5**. **Comparison of nested Cox proportional hazard models of time to incident cardiovascular disease**

Hazard ratios and 95% confidence intervals for Cox proportional hazard ratios for time to cardiac outcome for nested models. The full model refers to: Surv(tte, status) ~ Episcore + protein + age + sex, whereas the protein only model refers to Surv(tte, status) ~ protein + age + sex. Results are displayed for models where either predictor (measured protein or protein EpiScore) associated with incident cardiovascular disease at a nominal significance level (p<0.05). 10 of 17of the full models were significant (P_bonferonni_ < 0.05/17) and demonstrated an improved fit compared to a reduced model (likelihood ratio test, (P_bonferonni_ < 0.05/17)).

**Fig. S6 Distribution of Lambda for each iterative model in the OSCA marginal linear EWAS.** The dashed red line indicates a Lambda of 1.

**Fig. S7.** **Eigencor plot of the 20 included methylation principal components and other included covariates.** Values for pearson R are included, rounded to 2 significant figures.

**References**

1. Szklarczyk, D. *et al.* The STRING database in 2021: customizable protein-protein networks, and functional characterization of user-uploaded gene/measurement sets. *Nucleic Acids Res.* **49**, D605–D612 (2021).

2. Jr, F. E. H. *Hmisc: Harrell Miscellaneous*. (2023).

3. Gu, Z. Complex heatmap visualization. *iMeta* **1**, e43 (2022).
