# Supplementary material for: Methylome-wide association studies and epigenetic biomarker development for 133 mass spectrometry-assessed circulating proteins in 14,671 Generation Scotland participants
Source: Genome Biol. 2025 Dec 8;26:417. doi: 10.1186/s13059-025-03892-0 (PMC12683789; doi:10.1186/s13059-025-03892-0)
Supplement: Supplementary file 3 — Supplementary Material 3. [file 13059_2025_3892_MOESM3_ESM.docx]

**Supplementary Methods**

**M1. Mass Spec protein annotation**

Following data acquisition, processing and quality control [1,2], 439 inferred proteins were identified for 15,818 Generation Scotland participants. Signals were identified using an independent public spectral library [3] and annotated using UniProt [4,5]. The annotated signals were subsequently divided into uniquely mapped proteins (N = 133, 30.3%), where only one UniProt ID was identified and ‘protein groups’ (N = 306, 69.7%) where multiple UniProt IDs were assigned. The ‘protein groups’ could be further subdivided into groups containing variants of the same protein (N = 235), or groups containing mixed proteins (N = 75). For the purposes of this analysis, we used only the 133 uniquely mapped proteins (**Supplementary Table S15**).

**M2. GS DNA methylation QC**

Whole-blood DNA methylation data has been generated for 18,869 Generation Scotland participants. The full process has previously been described [6]. In brief, peripheral blood samples were collected in EDTA tubes and DNA extracted using Nucleon BACC3 extraction kits. DNA was Sodium bisulphite treated (EZ-96 DNA Methylation Kit (Zymo Research, Irvine, California) and methylation measured using the Illumina EPIC array and HiScan scanner. DNA methylation was profiled in four waves between 2016 and 2021. Quality review and control were carried out using GenomeStudio (version 2011.1) and in R using the packages: minfi (v.1.20.2 – 1.42.0); shinyMethyl v1.10.0, 1,14,0. 1,30.0 watermelon v.1.18.0, meffil vs. 1.1.0 and 1.1.2. Normalisation was carried out using the dasen method in the R package wateRmelon v.2.2.0 [7]. Methylation M-values were used for downstream analyses and were calculated using the beta2M function in the R package lumi v.2.30.0 [8]. M-values are the log2 ratio of intensities of methylated to unmethylated probe and are recommended for differential methylation analysis [9]. White cell proportions were estimated using either the Houseman algorithm via minfi or meffil [10,11].

**M3. Annotations of proteins and methylation sites**

A total of three proteins (A0A0G2JRQ6, A0A0J9YX35, A0A0J9YY99, all immunoglobulin-related proteins) could not be fully annotated to a genomic position in build37. Consequently, a total of 24 protein - CpG associations were not allocated to cis or trans effects. Both A0A0G2JRQ6 and A0A0J9YY99 are annotated to scaffolds HSCHR2_2_CTG7 and HSCHR14_3_CTG1 respectively. A0A0G2JRQ6, with 4 CpG associations, annotated to scaffold HSCHR2_2_CTG7 does not have an un-gapped mapping of its gene (Ensemble ID: ENSG00000281759, in build38) in the hg19 assembly. Similarly, A0A0J9YY99, with 13 CpG associations, on HSCHR14_3_CTG1 is not mapped in build37. Finally, A0A0J9YX35, with 7 CpG associations is annotated to the IGHV3-64D gene, which is annotated to HG1592_PATCH in build37. Therefore, these associations are counted in overall results but are not allocated to cis or trans effects.

**M4. EWAS catalogue search**

The catalogue download (on 05/08/25) was filtered to CpGs from the significant GMRMomi results (n = 697). It was further filtered to studies looking at “Whole blood” to match our biological sample, and to associations with a p value of < 3.6e-08 signifying genome-wide significance. All traits associated with these CpGs (n = 1015) were then searched using a combination of str_detect from the stringr package in R, for the UniProt ID, protein gene, the word “protein” and a visual inspection of all traits. A total of 13 previously reported associations were identified, all originating from work carried out in the Generation Scotland cohort, though with different protein data, and originate from two studies [12,13]. The study from Walker et. al., identifies DNAm differences between carriers of APOE e4 and e2 alleles and is therefore not directly comparable to measured protein levels [13]. Once this study is removed, 4 associations remained, entirely from Gadd et al.,’s Somascan study [12] (**Supplementary Table S7**).

**M5. GOmeth**

GOmeth function from the missMethyl package (version 1.38.0) in R was used to assess for enrichment of probe-associated gene sets [14]. This tool corrects for both probe-number and multi-gene bias in the methylation array data [14]. The top five pleiotropic proteins (P02748, P01834, O43866, P01742, P02750, assessed by highest numbers of trans associations), were taken forwards for analysis. For each protein, enrichment assessment was run for CpGs associated at PIP thresholds of 0.2, 0.4, 0.6, 0.8 and 0.95. The reference CpGs were taken to be the 752,722 CpGs from the EPIC array which passed QC in the GS dataset and were therefore part of the Bayesian analysis. Statistically significant results were taken to be those with a P_FDR_ < 0.05.

**M6. Definition of incident cardiovascular disease and censoring criteria for Cox Proportional Hazards models**

Disease occurrence data is available in Generation Scotland from linkage to secondary care NHS records with a total follow up period of up to 17.6 years from baseline appointment. A composite cardiovascular disease (CVD) outcome was defined as including any incidence of coronary heart disease, ischaemic stroke, myocardial infarction and cardiovascular-disease related death. In the independent test set within Generation Scotland (n = 3463), diagnoses were determined from secondary care records, using CALIBER/HDRUK consensus definitions[15,16]. CVD-related death was defined as described by Welsh et.al., [17] using the ICD-10 codes I00-I99, under the umbrella term “mortality from major cardiovascular diseases”. This includes deaths secondary to diseases of the heart (I00-I09, I11, I13, I20-I51), diseases related to hypertensive disorders (I10, I12, I15) and cerebrovascular diseases (I60-169). Any prevalent cases of CVD (as defined above), with a date of diagnosis prior to the initial GS appointment date, were excluded from the analysis (n = 116), a further two volunteers withdrew from the study between generation of the EpiScores and analysis of the linked data for the Cox proportional Hazard models, leaving a final test-set of 3345. Individuals were censored either at the end of the follow-up period (August, 2023) or on date of death if from a non-cardiovascular related cause, according to the ICD-10 codes specified above.

Additional covariates added to the models were determined as follows: prevalent diseases were ascertained through health-record linkage using secondary care data. Diseases were defined as prevalent if the diagnosis date was prior to the date of baseline blood-sample. The ICD codes used are detailed in Supplementary Table S25. Cholesterol levels (HDL and total) were established through laboratory analysis of baseline blood samples and systolic and diastolic blood pressures were recorded as the average of two measurements from the initial clinic visit.

**Bibliography**

1. Messner CB, Demichev V, Wendisch D, Michalick L, White M, Freiwald A, et al. Ultra-High-Throughput Clinical Proteomics Reveals Classifiers of COVID-19 Infection. Cell Syst. 2020;11:11-24.e4. https://doi.org/10.1016/j.cels.2020.05.012

2. Vernardis SI, Demichev V, Lemke O, Grüning N-M, Messner C, White M, et al. The Impact of Acute Nutritional Interventions on the Plasma Proteome. J Clin Endocrinol Metab. United States; 2023;108:2087–98. https://doi.org/10.1210/clinem/dgad031

3. Bruderer R, Muntel J, Müller S, Bernhardt OM, Gandhi T, Cominetti O, et al. Analysis of 1508 Plasma Samples by Capillary-Flow Data-Independent Acquisition Profiles Proteomics of Weight Loss and Maintenance. Mol Cell Proteomics MCP. United States; 2019;18:1242–54. https://doi.org/10.1074/mcp.RA118.001288

4. Soudy M, Anwar AM, Ahmed EA, Osama A, Ezzeldin S, Mahgoub S, et al. UniprotR: Retrieving and visualizing protein sequence and functional information from Universal Protein Resource (UniProt knowledgebase). J Proteomics. 2020;213:103613. https://doi.org/10.1016/j.jprot.2019.103613

5. The UniProt Consortium. UniProt: the Universal Protein Knowledgebase in 2023. Nucleic Acids Res. 2023;51:D523–31. https://doi.org/10.1093/nar/gkac1052

6. Walker RM, McCartney DL, Carr K, Barber M, Shen X, Campbell A, et al. Data Resource Profile: Whole-Blood DNA Methylation Resource in Generation Scotland (MeGS). Int J Epidemiol. 2025;54:dyaf091. https://doi.org/10.1093/ije/dyaf091

7. Pidsley R, Y Wong CC, Volta M, Lunnon K, Mill J, Schalkwyk LC. A data-driven approach to preprocessing Illumina 450K methylation array data. BMC Genomics. 2013;14:293. https://doi.org/10.1186/1471-2164-14-293

8. Du P, Kibbe WA, Lin SM. lumi: a pipeline for processing Illumina microarray. Bioinformatics. 2008;24:1547–8. https://doi.org/10.1093/bioinformatics/btn224

9. Du P, Zhang X, Huang C-C, Jafari N, Kibbe WA, Hou L, et al. Comparison of Beta-value and M-value methods for quantifying methylation levels by microarray analysis. BMC Bioinformatics. 2010;11:587. https://doi.org/10.1186/1471-2105-11-587

10. Aryee MJ, Jaffe AE, Corrada-Bravo H, Ladd-Acosta C, Feinberg AP, Hansen KD, et al. Minfi: a flexible and comprehensive Bioconductor package for the analysis of Infinium DNA methylation microarrays. Bioinforma Oxf Engl. England; 2014;30:1363–9. https://doi.org/10.1093/bioinformatics/btu049

11. Min JL, Hemani G, Davey Smith G, Relton C, Suderman M. Meffil: efficient normalization and analysis of very large DNA methylation datasets. Bioinformatics. 2018;34:3983–9. https://doi.org/10.1093/bioinformatics/bty476

12. Gadd DA, Hillary RF, McCartney DL, Shi L, Stolicyn A, Robertson NA, et al. Integrated methylome and phenome study of the circulating proteome reveals markers pertinent to brain health. Nat Commun. 2022;13:4670. https://doi.org/10.1038/s41467-022-32319-8

13. Walker RM, Vaher K, Bermingham ML, Morris SW, Bretherick AD, Zeng Y, et al. Identification of epigenome-wide DNA methylation differences between carriers of APOE ε4 and APOE ε2 alleles. Genome Med. 2021;13:1. https://doi.org/10.1186/s13073-020-00808-4

14. Maksimovic J, Oshlack A, Phipson B. Gene set enrichment analysis for genome-wide DNA methylation data. Genome Biol. 2021;22:173. https://doi.org/10.1186/s13059-021-02388-x

15. HDR UK CALIBER Phenotype Library [Internet]. https://portal.caliberresearch.org/

16. Kuan V, Denaxas S, Gonzalez-Izquierdo A, Direk K, Bhatti O, Husain S, et al. A chronological map of 308 physical and mental health conditions from 4 million individuals in the English National Health Service. Lancet Digit Health. Elsevier; 2019;1:e63–77. https://doi.org/10.1016/S2589-7500(19)30012-3

17. Welsh P, Preiss D, Hayward C, Shah ASV, McAllister D, Briggs A, et al. Cardiac Troponin T and Troponin I in the General Population. Circulation. United States; 2019;139:2754–64. https://doi.org/10.1161/CIRCULATIONAHA.118.038529
